# Physical Violence against General Practitioners and Nurses in Chinese Township Hospitals: A Cross-Sectional Survey

**DOI:** 10.1371/journal.pone.0142954

**Published:** 2015-11-16

**Authors:** Kai Xing, Mingli Jiao, Hongkun Ma, Hong Qiao, Yanhua Hao, Ye Li, Lijun Gao, Hong Sun, Zheng Kang, Libo Liang, Qunhong Wu

**Affiliations:** 1 Department of Health Policy and Hospital Management, School of Public Health, Harbin Medical University, Harbin, Heilongjiang province, China; 2 Harbin Medical University, Harbin, Heilongjiang province, China; 3 Endocrine and Metabolic Diseases 5 Wards, The 2nd Affiliated Hospital of Harbin Medical University, Harbin, Heilongjiang province, China; 4 Department of Social Medicine, School of Public Health, Harbin Medical University, Harbin, Heilongjiang province, China; 5 Department of Medical Demography, School of Public Health, Harbin Medical University, Harbin, Heilongjiang province, China; Örebro University, SWEDEN

## Abstract

**Purpose:**

The purpose of this study is to identify risk factors of physical violence in Chinese township hospitals.

**Methods:**

A cross-sectional survey was used in a sample of 442 general practitioners and 398 general nurses from 90 township hospitals located in Heilongjiang province, China (response rate = 84.8%).

**Results:**

A total of 106 of the 840 (12.6%) respondents reported being physically attacked in their workplace in the previous 12 months. Most perpetrators were the patients’ relatives (62.3%), followed by the patient (22.6%); 73.6% of perpetrators were aged between 20 and 40 years. Of the physical violence incidents, about 56.6% (*n* = 60) resulted in a physical injury, and 45.4% of respondents took two or three days of sick leave. Reporting workplace violence in hospitals to superiors or authorities was low (9.4%). Most respondents (62.8%) did not receive training on how to avoid workplace violence. Logistic regression analyses indicated that general nurses, aged 35 years or younger, and with a higher-level professional title were more likely to experience physical violence. Healthcare workers with direct physical contact (washing, turning, lifting) with patients had a higher risk of physical violence compared to other health care workers. Procedures for reporting workplace violence were a protective factor for physical violence; when in place, reporting after psychological violence (verbal abuse, bullying/mobbing, harassment, and threats) was more protective than waiting until an instance of physical violence (beating, kicking, slapping, stabbing, etc.).

**Conclusions:**

Physical violence in Chinese township hospitals is an occupational hazard of rural public health concern. Policies, procedures, and intervention strategies should be undertaken to manage this issue.

## Introduction

Workplace violence (WPV) toward healthcare workers in the healthcare sector has been recognized as a global problem and major public health concern[1–3]. Previous studies have shown that healthcare workers are more likely than other workers to be victims of violence or aggression[4–6]. Horizontal comparison is difficult when the definition of WPV was not fixed, violent workplace incidents that respondents needed to recall occurred at an earlier time, and the questionnaires used by different researchers varied. Nonetheless, even when these points were not considered, literature and studies from various countries showed that the percentage of healthcare workers suffering WPV was as high as 50% to 88%[7–11]. In China, the number of reports on WPV in hospitals has been increasing; a survey by Chen and Wang showed that 70.3% of doctors and 67.6% of nurses were victims of WPV in hospitals[12].

Regarding physical violence, between 1991 and 2001, there were 568 violent attacks reported relating to hospitals and healthcare workers in Hubei province. Between 2000 and 2002, there was an average of 177 such violent attacks each year in Jiangsu province. Even in Beijing, 502 violent attacks on healthcare workers were reported between 2000 and 2003[13]. In recent years, WPV, especially physical violence, in hospitals has become a serious social problem that affects social harmony and stability in China, and has caught the attention of both government and the general population. According to a report by the Chinese Hospital Association, the number of healthcare workers physically attacked has increased each year from 2008 to 2012; the proportion of hospitals where physical violence occurred has risen from 47.7% in 2008 to 63.7% in 2012. There was a six-fold increase in the proportion of physical violence occurring each year, and the proportion in 2012 (8.3%) was almost twice that of 2008 (4.5%)[14]. On October 25, 2013, three doctors from Wenling People’s Hospital in Zhejiang province were stabbed by a patient with a knife, resulting in one death and two injuries, which shocked the nation[15]. This is just one of many serious violent incidents in Chinese hospitals. Although non-physical violence can be just as detrimental as physical violence and is a part of the whole picture of workplace violence, non-physical violence was not included in this study after taking into account China’s current situation on physical violence.

The definition of WPV from the World Health Organization (WHO), which is used by researchers worldwide, includes physical violence and psychological violence[16]. Physical violence refers to physical attacks resulting in physical and psychological harm, including hitting, kicking, shooting, barring, pushing, biting, and other violent acts, such as sexual harassment and rape; psychological violence is the intentional act against the person or collective force that results in physical, mental, spiritual, moral, and social damages, including insults, threats, attacks, verbal abuse, and harassment[16]. This study only focused on the physical violence toward general physicians and nurses. Violence in the healthcare sector can normally result in adverse effects on healthcare workers, patients, and the efficiency and effectiveness of the health systems. It includes direct costs, such as illness, disability, death, lost work days, staff shortage, and loss of property; and indirect costs, such as increased occupation stress and security costs, decreased motivation and job satisfaction, reduced work performance, poor patient care quality, recruitment issues, and lower creativity levels[17–21]. More seriously, violence in the healthcare sector has begun to affect the next generation of healthcare workers in China. In 2001, according to the Chinese Medical Doctors’ Association, 11% of doctors hoped their own children would choose the medical profession in the future; this had dropped to 7% in 2011[22].

Although most previous research has focused on general hospitals in cities, WPV in the healthcare sector in rural areas has already been studied in several other countries. Nonetheless, Hegney and colleagues[23] found policies and procedures for WPV lacking in rural and remote areas; this is despite Tolhurst and colleagues[24] identifying WPV against rural general practitioners (GPs) as a frequent problem. Magin and colleagues[25] reported that GPs in rural practices were more likely than those in urban practices to have experienced violence; Meuleners and colleagues[26] reported that rural and remote GP practices reported a higher incidence of violent presentations compared to their metropolitan counterparts. Safe facilities for after-hours care and training for GPs in managing dangerous situations should be provided. In particular, high-risk groups, such as women and those living in rural and remote areas, should be targeted for special attention. However, few studies have investigated the risk factors for violence in Chinese township hospitals.

In China, primary health care institutions include community health service centers, township hospitals, village clinics and outpatient clinics. Community health service centers are comprehensive health administration and medical institutions providing basic health services, including prevention, treatment, rehabilitation and health promotion for community residents living in urban areas.Township hospitals, village clinics and outpatient clinics are comprehensive health administration and medical institutions providing basic rural health services for people living in the towns and villages. Township hospitals are regarded as the hubs of the rural tertiary healthcare system. China’s new round of healthcare reform and its twelfth Five-Year Plan for the medical service system focus on activities to improve and strengthen town-level health facilities. The goal is to reduce common and frequently occurring diseases. GPs are central to the health personnel teams of township hospitals in the future because of the status and working characteristics of township hospitals in China[27]. According to China’s Ministry of Health report “China Health Statistics Yearbook 2013”[28], of the 37,097 township hospitals in China, 996 were in Heilongjiang province, and these had 2,081 GPs and 3,616 registered nurses.

The current study purposes are to identify the prevalence and level of severity of physical violence against general practitioners and nurses in township hospitals in Heilongjiang province, northeastern China, and to identify the risk factors contributing to physical violence in Chinese township hospitals.

## Materials and Methods

A cross-sectional survey was conducted in Heilongjiang province, China. In 2012, Heilongjiang had a population of 38.1 million with 996 township hospitals. The study was in three locations: Harbin,Daqing and Hegang. Due to the study’s time and resource limitations, we purposively selected 90 township hospitals (approximately 10%) according to geographical location, populations, and health levels of each township. This comprised 30 hospitals in the Harbin area (middle), 30 hospitals in the Daqing area (west), and 30 hospitals in the Hegang area (east). Permission was obtained from all 90 township hospitals.

### Data collection

The survey was conducted from September to November 2014, and access was negotiated through supervisors in every study hospital. An anonymous, self-administered questionnaire was distributed to each participant. A notification letter and return envelope were included with each questionnaire, and a box was kept in the department manager’s office. The study purpose and rights of the healthcare workers regarding participation were declared in the letter. The participants had 7 days to complete the questionnaire; then they were asked to return the completed questionnaire in the envelope and put the envelope into the prepared box in the department manager’s office to ensure privacy and anonymity. The data collected were secured in a locked room that could only be accessed by research personnel. A total of 990 questionnaires were distributed, and 840 valid questionnaires were obtained (response rate = 84.8%).

### Questionnaire

The questionnaire used was developed based on a literature review and a modified version of the questionnaire developed in 2003 by the International Labour Office (ILO), International Council of Nurses (ICN), WHO, and Public Services International (PSI) joint program[29]. First, we formally obtained documented permission to use the questionnaire from the ILO and WHO; then, the questionnaire was modified to fit the objectives of the study and the township hospital context in China. It was then translated into Chinese. Its content validity was examined by 18 healthcare-related experts throughout China; they were asked to assess the questionnaire for its clarity, relevancy, comprehensiveness, and sensitivity to China’s culture. The questionnaire was then pre-tested with 30 participants who were subsequently excluded from the study. In accordance with the participants’ feedback, further modifications were undertaken. For allquestions, Cronbach’s alpha coefficients for all the scales were 0.86. The questionnaire was then back translated to English to verify the accuracy of the Mandarin version.

We deleted, modified, and added some items to the original questionnaire. In order to fit the study aims and Chinese culture, we deleted eight items such as “Did you move from another country to the place where you are currently working?”, modified four items such as age, occupation, professional title and added three new items about training in managing aggression and violence in the first section. In the second section, we deleted one item “Which day of the week did it happen?”, modified three items such as “Where did the incident take place?” and added four items such as the ages and genders of the perpetrators. In the third section, we put five types of psychological violence into one table, integrated five separate parts into one part and modified one item “Where did the psychological violence take place?”. We also integrated two items into one item and modified this to adapt to the current situation on WPV in China.

Therefore, the modified questionnaire has four sections: (1) the demographic characteristics of the respondents and workplace data; (2) physical violence, including prevalence of physical violence, demographic characteristics, attack time, attack tools, consequences for the perpetrators, and so on; (3) mental and emotional violence; and (4) organizational measures, including incident reporting, supervisor support, training programs, and so on. Because this study is only about physical violence, the data needed were only taken from Sections 1, 2, and 4.

### Data analysis

We coded the data in EpiData, and asked another member of our team to verify all entered data. The data were analyzed using IBM SPSS Statistics 19.0 (Statistical Product and Service Solutions 19.0, 2010, www.spss.com). Analyses began with basic descriptive statistics regarding the characteristics of the perpetrators and victims in the hospital violence. Binary logistic regression analysis was used to assess potential associations between exposure to violence in general (yes/no) and respondents’ characteristics, including age, gender, years of experience, educational level, occupation, professional title, and direct physical contact (washing, turning, lifting) with patients as well as procedures for reporting violence and training to manage aggressive or violent situations. Through the gradually filtering method of logistic regression (criteria of variable selection: independent variables were entered and excluded from the logistic regression model at *p* < 0.05), age, occupation, professional title, direct physical contact (washing, turning, lifting) with patients, and procedures for reporting violence were used in the final binary logistic regression model. Odds ratios (OR) and 95% confidence intervals (CI) were calculated; *p* < 0.05 was considered statistically significant.

### Ethical approval

Ethical approval was granted by the Institutional Review Board of Harbin Medical University before data collection commenced, and all procedures were approved by each study hospital. All participants gave their informed consent to participate.

## Results

A total of 840 out of 990 questionnaires were returned (response rate = 84.8%) from 442 GPs and 398 general nurses. Only valid responses and percentages were included. Both descriptive and binary logistic regression analysis results are presented below.

### Demographic characteristics of the respondents

A summary can be seen in Table 1.

**Table 1:** Demographic characteristics of the respondents (*N* = 840).

Characteristics	*n*	%
**Gender**		
Male	442	52.6
Female	398	47.4
**Age**		
≤35	140	16.7
35–45	426	50.7
≥45	274	32.6
**Years of experience**		
1–10	116	13.8
11–20	394	46.9
21–30	294	35.0
>30	36	4.3
**Education**		
Postgraduate	8	1.0
Undergraduate	380	45.2
College	348	41.4
Technical secondary school education and below	104	12.4
**Professional title**		
Senior	170	20.2
Intermediate	410	48.8
Junior	192	22.9
No title	68	8.1
**Occupation**		
General practitioner	442	52.6
General nurse	398	47.4

### Prevalence of physical violence and perpetrators

Physical violence was experienced in the previous 12 months by 12.6% of the questionnaire respondents (*n* = 106). Respondents reported patients’ relatives as the main aggressors (62.3%, *n* = 66), followed by patients (22.6%, *n* = 24). A total of 62.3% (*n* = 66) of physical assaults were conducted with knives and sticks prepared in advance or with furniture, such as tables and chairs, in the ward. Regarding consequences for the perpetrators, 50.9% of the victims chose “none” followed by a “verbal warning issued by hospital managers” (26.4%) (Table 2).

**Table 2:** Prevalence (%) and characteristics of physical violence in township hospitals of Heilongjiang province, and consequences for the perpetrators.

	*n*	%
**Exposure to physically violent incidents in the last 12 months**		
Yes	106	12.6
No	734	87.4
**Perpetrators**		
Patient	24	22.6
Relatives of patient	66	62.3
General public	16	15.1
**Gender of the perpetrators**		
Male	94	88.7
Female	12	11.3
**Age of the perpetrators**		
<20	16	15.1
20–40	78	73.6
40–60	12	11.3
**Attack time**		
After drinking	20	18.9
After patient took medicine	28	26.4
When patient suffered disease progression	58	54.7
**Attack tool**		
Knives and sticks prepared in advance	36	34.0
Furniture in the ward, such as tables and chairs	30	28.3
No	40	37.7
**Consequences for the perpetrators**		
None	54	50.9
Verbal warning issued by hospital managers	28	26.4
Care discontinued	0	0
Reported to police	24	22.7
Aggressor prosecuted	0	0

### Victims and effect of physical violence on victims

Of the 106 victims, most victims were female (60.4%) and general nurses (62.3%). Of the physical violence incidents, about 56.6% (*n* = 60) resulted in a physical injury. About 62.3% of victims took time off work due to an assault; of these, 45.4% were off work for two to three days. Of those who did not report the events, the top three reasons were “it is useless” (69.8%), “afraid of negative consequences” (58.5%), and “felt ashamed” (41.5%). A total of 53.8% of victims considered these violent incidents to be preventable (Table 3).

**Table 3:** Characteristics, cognitive and behavioral response of the victims, and influence of physical violence on victims (*n* = 106).

	*n*	%
**Gender of victims**		
Male	42	39.6
Female	64	60.4
**Age of victims**		
≤35	28	26.4
35–45	46	43.4
≥45	32	30.2
**Occupation of victims**		
General practitioner	40	37.7
General nurse	66	62.3
**Injured as a result of the physical violence**		
Yes	60	56.6
No	46	43.4
**Time off from work after being attacked**		
Yes	66	62.3
No	40	37.7
**Duration of time off after the physical violence**		
1 day	20(66)*	30.3
2–3 days	30	45.4
1 week	12	18.2
2–3 weeks	4	6.1
**Response to the incident** ^**1**^		
Told the person to stop	37	34.9
Tried to defend myself physically	74	69.8
Confiding in friends/family	14	13.2
Sought counseling	8	7.5
Told a colleague	32	30.2
Transferred to another position	16	15.1
Reported it to a senior staff member	22	20.8
Completed incident/accident form	16	15.1
Pursued prosecution	8	7.5
Completed a compensation claim	8	7.5
Sought help from the union	0	0
**Reason for not reporting the incidents** ^**1**^		
It was not important	40	37.7
Felt ashamed	44	41.5
Felt guilty	28	26.4
Afraid of negative consequences	62	58.5
Useless	74	69.8
Did not know who to report to	41	38.7
**Incident could have been prevented**		
Yes	57	53.8
No	49	46.2

(^1^): multiple choice

*:66 means that 66 victims took time off work due to an assault

### Policies, procedures, and intervention strategies of employers

A total of 47.4% of respondents (*n* = 398) said that in their workplace they did not have procedures for reporting violence. When there was a reporting system, 58.4% (*n* = 258) knew how to use it. A total of 54.6% (*n* = 459) said that there was no encouragement to report workplace violence. Only 37.3% reported having training in managing aggression and violence. A total of 90.6% respondents (*n* = 761) in the past year had not reported physical violence (Table 4).

**Table 4:** Policies, procedures, and intervention strategies against workplace violence.

	*n*	%
**Procedures for reporting workplace violence**		
Reporting only if suffered physical injury	258	30.7
Reporting if suffered verbal threat or injuries	184	21.9
No	398	47.4
**If have procedures, know how to use them**		
Yes	258(442)	58.4
No	184	41.6
**Encouragement to report workplace violence**		
Yes	381	45.4
No	459	54.6
**Interventions taken by employer or supervisor**		
Counseling	78(106)	73.6
Other support	47(106)	44.3
**Reporting incidents of workplace violence in past 12 months (witnessed or experienced)**		
Yes	79	9.4
No	761	90.6
**Training in managing aggression and violence**		
Yes	176	37.2
No	297	62.8

### Binary logistic regression analysis

Age, occupation, and professional title of respondents had significant associations with exposure to physical violence in general (yes/no). There was a lower risk of physical violence for respondents who were 35 years and older. Compared to GPs, the occupation at a higher risk for physical violence was general nurses; those with a lower level of professional title were less likely to be victims of physical violence compared to their counterparts. In addition, direct physical contact with patients increased the risk of physical violence. Procedures for reporting workplace violence were a protective factor for physical violence. When such procedures are in place, reporting when one has suffered psychological violence (verbal abuse, bullying/mobbing, harassment, and threats) is more protective than waiting until instances involve physical violence (beating, kicking, slapping, stabbing, etc.) (Table 5).

**Table 5:** Risk factors associated with physical violence among general practitioners and general nurses in township hospitals in Heilongjiang province; binary logistic regression model results.

		Physical aggression (*n* = 106)			
		*n*	%	Adjusted ORs	95% CIs
**Age**	≤35	28	26.4	1	
	35–45	46	43.4	0.461**	(0.263, 0.809)
	≥45	32	30.2	0.539*	(0.295, 0.984)
**Occupation**	General practitioner	40	37.7	1	
	General nurse	66	62.3	2.739***	(1.735, 4.322)
**Professional title**	Senior	34	32.1	1	
	Intermediate/Junior/No title	72	67.9	0.622***	(0.483, 0.800)
**Direct physical contact (washing, turning, lifting) with patients**	Yes	80	75.5	1	
	No	26	24.5	0.411***	(0.247, 0.685)
**Procedures for reporting violence in your workplace**	Reporting when one has suffered psychological violence	29	27.3	0.478**	(0.292, 0.782)
	Reporting until instances involve physical violence	4	3.8	0.088***	(0.031, 0.248)
	No	73	68.9	1	

*p < 0.05

*p < 0.01

***p < 0.001.

## Discussion

Previous research has found that age was a risk factor for workplace violence in general hospitals[20,30,31]. Our findings indicated that respondents 35 years or younger experienced higher levels of physical violence in township hospitals. Their increased risk may be because they have little work experience and opportunities to improve, possibly making some mistakes and errors. When this happens, they may have fewer healthy communication skills with patients. Furthermore, respect for elders is considered a traditional virtue in China, which has been around for thousands of years: this refers to not only respect for one’s own elders, and but also respect other people's elders[32]. Under the influence of Chinese traditional culture, older healthcare workers were less likely to suffer physical violence compared to those who were younger.

In addition, there was an increased likelihood of experiencing physical violence among healthcare workers with a higher professional level. This may be because the higher the level of the healthcare workers, the greater workload they have; the high intensity of the work not only affects the health of healthcare workers, but also affects the quality of the medical services provided. For example, it may result in poor service attitude, misdiagnosis, and so forth, further increasing the likelihood of workers experiencing physical violence. According to a report of the Chinese Medical Doctors’ Association, the higher the level of physician, the greater proportion of overtime being worked; 94% of professional doctors with a senior title must work overtime and 40% of professional doctors basically cannot take annual leave[21].

Previous research has found that nursing was the healthcare occupation at the highest risk for workplace violence[33–38]. Our analyses also indicated that general nurses were most likely to experience physical violence in township hospitals. This may be because nurses are more likely to have direct physical contact with patients through their daily tasks, such as giving medication and drawing blood samples. In addition, according to the report “China Health Statistics Yearbook 2013” by China’s Ministry of Health[28], the proportion of doctors and nurses in township hospitals in Heilongjiang province is 2.26:1; therefore, nurses in township hospitals may have a greater workload compared to doctors in township hospitals, and may have a higher risk of experiencing physical violence. Additionally, those who were more likely to have direct physical contact with patients were more likely to be at a higher risk for physical violence, which is consistent with the results of previous studies[20,34,39,40].

Our surveys indicated that procedures for reporting violence were a protective factor for violent incidents. However, many previous studies on WPV showed that under-reporting of violence to hospital superiors or authorities was a concern[41–44]. Only 9.4% of the respondents in our study indicated having reported an incident of workplace violence to hospital superiors or authorities in the last 12 months. This may be related to the lack of incident reporting policies in the township hospital system; in our system, 47.4% of healthcare workers indicated an absence of procedures for reporting violence. Therefore, it is necessary for township hospitals to develop reporting systems for WPV. Another reason for this under-reporting may be misperceptions by healthcare workers about reporting the incident to hospital superiors or authorities. The study results suggested that the top three reasons for not reporting were that it was “useless,” they were “afraid of negative consequences,” and they “felt ashamed”. This is similar to the findings of Kitaneh and Hamdan[45] and reflects that a reporting system alone is not enough;organizational support is also very important. The results of Findorff and colleagues’ study suggested that supervisor support in the work environment was protective for physical and psychological violence[39]. Flannery and colleagues[46]found that support after a violent incident can lead to a reduction in future violence. Therefore, hospitals should encourage their staff to report WPV. At the same time, when violence happens, counseling and other kinds of professional support should be made available to victims. Yet, in this study, a disappointing 54.6% of respondents believed that they were not encouraged to report WPV. Meanwhile, although 73.6% of the victims indicated that their employer or supervisor offered counseling, only 55.7% indicated that their employer or supervisor offered other support. Therefore, hospitals need to improve their efforts in these areas.

When procedures for reporting violence are in place, reporting when one has suffered psychological violence (verbal abuse, bullying/mobbing, harassment, and threats) is more protective than waiting until instances involve physical violence (beating, kicking, slapping, stabbing, etc.). Crisis management theory, proposed by Robert Heath, suggests that the sooner managers prepare to deal with a crisis, the smaller the ramifications of the crisis; therefore, managers of township hospitals should develop an early warning system for crises to assess the potential risks and minimize possible hazards[47]. At the same time, the present study only focuses on physical violence; further research on psychological violence must be undertaken in the future.

Similar to other studies[31,48,49], this study demonstrated that physical violence was mainly initiated by patients’ relatives, followed by the patients themselves. The reason for this could be the closeness of their relationship. The results indicated that male perpetrators aged between 20 and 40 years, and perpetrators suffering disease progression or who had received bad news from patients appeared more likely to become aggressive. Patients experiencing disease progression may have difficulty accepting their reality, which may trigger aggressive behavior. This is the same for the patients’ relatives. Healthcare workers should understand the mood of patients and their relatives, and respond with empathy, trying to establish a safe atmosphere by consoling them. Patient autonomy and the individual character of patients and their relatives also need to be considered.

The influence of physical violence on healthcare workers is an issue of concern. Physical injuries in the healthcare sector can result in serious physical damage. According to our findings, 34.0% of physical violence occurred with knives and sticks prepared in advance, while 28.3% occurred with furniture in the ward, such as tables and chairs. A total of 56.6% of victims were injured as a result of violent incidents; 62.3% of victims took leave due to an assault, which is a much higher result compared to previous findings[50,51]. Thus, we recommend several precautionary measures to deal with this issue. For example, everyone who is not a hospital employee should receive mandatory inspections for any dangerous implements before entering the hospital; furniture in the ward, such as tables and chairs, should be placed in their proper position and be fixed to ensure that they cannot be moved freely; and healthcare workers can equip themselves with protective equipment, such as batons and helmets. A study reported that 16% of physicians in healthcare settings in Michigan, USA, carried a concealed weapon as a form of protection because they feared workplace violence or had previously experienced such violence[52].

One of the important findings in this study is regarding the consequences for the perpetrators; 50.9% of the perpetrators were not subject to any form of punishment. To some extent, this reflects a lack of mandatory policies and legislation concerning the punishment of perpetrators. No specific laws to protect the basic personal safety of healthcare workers and punish perpetrators have existed until now in China. Therefore, we suggest that the government accelerate the implementation of health and safety legislation. Meanwhile, rather than inaction, hospitals should take countermeasures regarding the punishment of perpetrators.

According to the WHO, in general, violence in society is preventable[16]. Indeed, our findings indicated that 53.8% of victims considered the event preventable; this is in line with the the conclusion of the WHO. Therefore, after identifying and understanding the risk factors contributing to physical violence, a substantial proportion of physical violence could be avoided through various interventions.

Although training in managing aggression and violence was not statistically significant in this study, it is somewhat alarming that 62.8% of the staff reported having not received training in managing aggression and violence. As training is an effective way to avoid and reduce workplace violence[6,30,53,54]. Therefore, we recommend that the township hospitals provide training programs for staff in dealing with and avoiding workplace violence.

### Limitations

The present study has several limitations. First, due to time and resource restrictions, our study was limited to one province in China and to only 90 purposively selected township hospitals in Heilongjiang province; therefore, we cannot generalize our findings to all of the township hospitals in Heilongjiang province and across China. However, our findings can provide a guide for further research on WPV in Chinese township hospitals. Second, recall bias may have existed due to the data collection method, which involved questionnaires and participants needing to recall events in the last 12 months. Third, the survey we conducted was organized by individual rather than government agencies, so we can only obtain participants’ views on workplace violence in their workplace, such as through the question “Are there procedures for the reporting of violence in your workplace?”. However, we cannot know whether township hospitals actually have procedures in place for the reporting of violence. This is a limitation of present study. Nonetheless, we think that even if township hospitals have procedures for the reporting of violence, but the hospital staff did not know about the existence of such policies, then that is equivalent to having no procedures for the reporting of violence. Fourth, this study only focused on physical violence; further studies on psychological violence are needed.

## Conclusion

This study is one of the rare reports concerning physical violence against GPs and general nurses in Chinese township hospitals. Physical violence in Chinese township hospitals as an occupational hazard is of rural public health concern. The findings in this study indicate that a series of measures should be implemented to deal with physical violence, including the development or improvement of policies, procedures, and intervention strategies. Further research into psychological violence should also be undertaken.

## Supporting Information

S1 Appendix Workplace Violence in the Health Sector Case Study—Questionnaire. This is the questionnaire we used in our study.
(DOC)

## Abbreviations

WPV: Workplace violence
WHO: World Health Organization
GPs: General practitioners
