# Supplementary material for: Physical Violence against General Practitioners and Nurses in Chinese Township Hospitals: A Cross-Sectional Survey
Source: PLoS One. 2015 Nov 16;10(11):e0142954. doi: 10.1371/journal.pone.0142954 (PMC4646672; doi:10.1371/journal.pone.0142954)
Supplement: S1 Appendix — This is the questionnaire we used in our study. (DOC) [file pone.0142954.s001.doc]

**Workplace Violence in the Health Sector**

**Case Study--Questionnaire**

***Please complete the questionnaire by either ticking boxes or writing in the spaces provided. If you don’t know how to answer one question, just go on to the next one.***

**A. PERSONAL AND WORKPLACE DATA**

**A 1** Age:

**A 2** Gender: 🞏 Male 🞏 female

**A 3** what is your Marital status: 🞏 Single 🞏 Married 🞏 living with partner

🞏 Separated/ Divorced 🞏 widow/Widower

**A 4** How many years of work experience in the health sector do you presently have: _______

**A 5** Education: 🞏Postgraduate 🞏Undergraduate 🞏College 🞏Technical secondary school education and below

**A 6** Ethnic group：

**A 7** Professional title: **🞏** Senior **🞏** Intermediate **🞏** Junior **🞏** No title

**A 8** Occupation: **🞏** Doctor department **🞏** Nurse department

**A 9** Company name______________ __

**A 10** Do you work in shifts? 🞏 Yes 🞏 No

**A 11** Do you work anytime between 18h00 (6 PM) and 07h00 (7 AM)? 🞏 Yes 🞏 No

**A 12** Do you interact with patients/clients during your work? No*, please go to question* ***A 14***

**12.1.** Do you have routine direct physical contact (washing, turning, lifting) with

patients/clients? 🞏 Yes 🞏 No

**12.2.** The patients/clients you most frequently work with are (*tick all appropriate boxes*):

🞏 Newborns/Infants 🞏 Children/Adolescents (10-18 years of age) 🞏 Adults 🞏 Elderly

**12.3.** The sex of the patients you most frequently work with are：

🞏 Male 🞏 Female 🞏 Male and female

**A 13** How worried are you about violence in your current workplace? (*Please rate:* 1 = not worried at all; 5 = very worried):

🞏1 🞏2 🞏3 🞏4 🞏5

**A 14** Are there procedures for the reporting of violence in your workplace? *If NO, please go to question* ***A 17***

🞏 Reporting only if suffered physical injuries 🞏 Reporting if suffered verbal threats or injuries 🞏 No

**14.1.** If YES, do you know how to use them? 🞏 Yes 🞏 No

**A 15** Is there encouragement to report workplace violence?*If NO, please go to next section*

🞏 Yes 🞏 No

**15.1.** If YES, by whom : 🞏 Management / Employer 🞏 Colleagues

🞏 Union or association 🞏 Own family / friends 🞏 Other, *please specify:________*

**A 16** have you received training in managing aggression and violence? 🞏 Yes 🞏 No (*If NO, please go to question* ***A 18***)

**A 17** How many times have you received training in managing aggression and violence?_____ times

**A 18** Are you willing to participate in the training?（*Please rate: 1 = very unwilling, 5 = very*

willing） 🞏1 🞏2 🞏3 🞏4 🞏5

**B. PHYSICAL WORKPLACE VIOLENCE**

**Physical violence refers to the use of physical force against another person or group, that results in physical harm, sexual or psychological harm. It can include beating, kicking, slapping, stabbing, shooting, pushing, biting, and/or pinching, among others.**

**B 1 In the last 12 months**, have you been physically attacked in your workplace? If No*, please go to question* ***B 2***

🞏 Yes， times 🞏 No

**1.1.**Some information on the last time you have been physically attacked in your workplace：

**1.1.1** Age of the perpetrators：🞏20 or under 🞏20-40 🞏40-60

**1.1.2** Gender of the perpetrators：🞏 Male 🞏 Female

**1.1.3** Attack time：🞏 After drinking 🞏 After patient took medicine

**1.1.4** If perpetrators were patients ： 🞏 suffered disease progression

**1.1.5** Is there a weapon：

🞏 Knives and sticks prepared in advance 🞏Furniture in the ward, such as tables and chairs 🞏 No

**1.1.6** The biggest role is to stop events：

🞏 Own 🞏 Colleague 🞏 Other patients and relatives

🞏 The hospital security guards 🞏 Police

**1.1.7** You think the cause of the incident including：

🞏 Negative media guide 🞏 Mental disorders of patients 🞏 Colleague instigated

🞏 Presence of gang members 🞏 Poor communication 🞏 The requirements of the patients or relatives did not meet 🞏 High medical expenses paid by own

🞏 Patient died after After the rescue invalid

🞏 No improvement in the patient's condition or no improvement since that

🞏 Long waiting time 🞏 Seek financial compensation

🞏 Not satisfied with the doctor work 🞏Not satisfied with the nurse work

🞏 Other, *please specify*

**1.2.** Do you consider this to be a typical incident of violence in your workplace?

🞏 Yes 🞏 No

**1.3.** Who attacked you?**：**🞏 Patient 🞏 Relatives of patient 🞏 Staff member

🞏 Management / Supervisor 🞏 External colleague/worker

🞏 General public 🞏 Other, *please specify:*

**1.4.** Where did the incident take place?：🞏 The emergency room 🞏 Ward

🞏 The corridor 🞏 Outpatient department 🞏 The doctor's office

🞏 The nurse station 🞏 Treatment room 🞏 On the way to the office

🞏 Hospital cafeteria 🞏 The parking lot 🞏 Other_____

**1.5.** At which time did it happen? 🞏 Don’t remember 🞏 07.00h.- before 13.00 h.

🞏 18.00h. – before 24.00 🞏 24.00h-before 07.00h

**1.6.** How did you respond to the incident? （*Please tick all relevant boxes*）

🞏 Took no action 🞏 Tried to pretend it never happened

🞏 Told the person to stop 🞏 Tried to defend myself physically

🞏 Confiding in friends/family 🞏 Sought counseling 🞏 Told a colleague

🞏 Transferred to another position 🞏 Reported it to a senior staff member

🞏 Completed incident/accident form 🞏 Pursued prosecution

🞏 Completed a compensation claim 🞏 Sought help from association and union

**1.7.** Do you think the incident could have been prevented? 🞏 Yes 🞏 No

**1.8.** Were you injured as a result of the violent incident? *if NO, please go to question* ***1.9***

🞏 Yes 🞏 No

**1.8.1.** IF YES, did you require formal treatment for the injuries? 🞏 Yes 🞏 No

**1.9. Listed below are a list of problems and complaints that people sometimes have in response to stressful life experiences like the event that you suffered. *For each item, please indicate how bothered you have been by these experiences since you were attacked. Please tick one option per question.***

| Since you were attacked, how **BOTHERED** have you been by: | Not at All | A Little Bit | Moderately | Quite a Bit | Extremely |
| --- | --- | --- | --- | --- | --- |
| Repeated, disturbing memories, thoughts, or images of the attack? | 🞏 | 🞏 | 🞏 | 🞏 | 🞏 |
| Avoiding thinking about or talking about the attack or avoiding having feelings related to it? | 🞏 | 🞏 | 🞏 | 🞏 | 🞏 |
| Being "super-alert" or watchful and on guard? | 🞏 | 🞏 | 🞏 | 🞏 | 🞏 |
| Feeling like everything you did was an effort? | 🞏 | 🞏 | 🞏 | 🞏 | 🞏 |

**1.10.** Did you have to take time off from work after being attacked? *if NO, please go to question* ***1.11.***  🞏 Yes 🞏 No

**1.10.1.** If YES, for how long? 🞏 One day 🞏 2-3 days 🞏 One week 🞏 2-3 weeks 🞏 1 month

🞏 2-6 months 🞏 7-12 months

**1.11.** Was any action taken to investigate the causes of the incident? *IF NO or DON’T KNOW please go to question* ***1.12***

🞏 Yes 🞏 No 🞏 Don’t know

**1.11.1.** IF YES, by whom: 🞏management / employer 🞏union 🞏association 🞏police

🞏other, *please specify:______________*

**1.11.2.** What were the consequences for the attacker?

🞏 None 🞏 Verbal warning issued by hospital managers 🞏 Care discontinued 🞏 Reported to police

🞏 Aggressor prosecuted 🞏 Other:____ 🞏 Don't know

**1.12.** Did your employer or supervisor offer to provide you with:

**1.12.1** Counseling 🞏 Yes 🞏 No

**1.12.2** Opportunity to speak about/report it 🞏 Yes 🞏 No

**1.13.3** Other support? 🞏 Yes 🞏 No

**1.13.** How satisfied are you with the manner in which the incident was handled? *(Please rate: 1 = very dissatisfied, 5 = very satisfied)*

🞏1 🞏2 🞏3 🞏4 🞏5

**1.14.** If you did **not** report or tell about the incident to others, why not? **（***Please tick every relevant box***）**

🞏 It was not important 🞏 Felt ashamed 🞏 Felt guilty

🞏 Afraid of negative consequences 🞏 Useless

🞏 Did not know who to report to 🞏 Other, *please specify*:__________

**B 2 In the last 12 months,** have you witnessed incidents of physical violence in your workplace? *if NO, please go to question* ***B 3*** 🞏 Yes 🞏 No

**2.1.** If YES, how often has this occurred in the last 12 months? 🞏 Once 🞏 2-4 times

🞏 5-10 times 🞏 Several times a month 🞏 About once a week 🞏 Daily

**B 3** Have you reported an incident of workplace violence **in the last 12 months**? (witnessed or experienced) *If NO, please go to section: PSYCHOLOGICAL VIOLENCE*

🞏 Yes 🞏 No

**3.1** IF YES, have you been disciplined for reporting an incident of workplace violence?

🞏 Yes 🞏 No

**C.PSYCHOLOGICAL WORKPLACE VIOLENCE**

**Psychological violence is defined as: Intentional use of power, including threat of physical force, against another person or group, that can result in harm to physical, mental, spiritual, moral or social development. Psychological violence includes verbal abuse, bullying/mobbing, harassment, and threats.**

**C 1 In the last 12 months**, have you suffered psychological violence in your workplace?

🞏 Yes 🞏 No (*if NO, please go to* ***section D****)*

|  | all the time | sometimes | once |
| --- | --- | --- | --- |
| Verbal abuse | 🞏 | 🞏 | 🞏 |
| Bullying/Mobbing | 🞏 | 🞏 | 🞏 |
| Threats | 🞏 | 🞏 | 🞏 |
| Verbal sexual harassment | 🞏 | 🞏 | 🞏 |
| Sexual harassment | 🞏 | 🞏 | 🞏 |

**C 2** Please think of **the last time** you suffered psychological violence in your place of work. By who?

🞏 Patient 🞏 Relatives of patient 🞏 Staff member

🞏 Management / Supervisor 🞏 External colleague/worker

🞏 General public 🞏 Other, *please specify:*

**C 3** Do you consider this to be a typical incident of psychological violence in your workplace?

🞏 Yes 🞏 No

**C 4** Where did the psychological violence take place?:

🞏 The emergency room 🞏 Ward 🞏 The corridor

🞏 Outpatient department 🞏 The doctor's office 🞏 The nurse station 🞏ICU

🞏 Treatment room 🞏 On the way to the office 🞏 Hospital cafeteria

🞏 The parking lot 🞏 On the phone 🞏 Other_____

**C 5** How did you respond to the psychological violence? (*Please tick all relevant boxes)*

🞏 Took no action 🞏 Tried to pretend it never happened

🞏 Told the person to stop 🞏 Tried to defend myself physically

🞏 Told friends/family 🞏 Sought counseling 🞏 Told a colleague

🞏 Transferred to another position 🞏 Reported it to a senior staff member

🞏 Completed incident/accident form 🞏 Pursued prosecution

🞏 Completed a compensation claim 🞏 Sought help from association and union

**C 6** Listed below are a list of problems and complaints that people sometimes have in response to stressful life experiences like the event that you suffered. *For each item, please indicate how bothered you have been by these experiences since you suffered psychological violence . Please tick one option per question.*

| Since you were abused, how **BOTHERED** have you been by: | Not at All | A Little Bit | Moderately | Quite a Bit | Extremely |
| --- | --- | --- | --- | --- | --- |
| Repeated, disturbing memories, thoughts, or images of the attack? | 🞏 | 🞏 | 🞏 | 🞏 | 🞏 |
| Avoiding thinking about or talking about the attack or avoiding having feelings related to it? | 🞏 | 🞏 | 🞏 | 🞏 | 🞏 |
| Being "super-alert" or watchful and on guard? | 🞏 | 🞏 | 🞏 | 🞏 | 🞏 |
| Feeling like everything you did was an effort? | 🞏 | 🞏 | 🞏 | 🞏 | 🞏 |

**C 7** Do you think the incident could have been prevented? 🞏 Yes 🞏 No

**C 8** Was any action taken to investigate the causes of the psychological violence ? *If NO or DON’T KNOW, please go to question* ***C* 9**

🞏 Yes 🞏 No 🞏 Don’t know

**8.1.** If YES, by whom: *(please tick every relevant box)*

🞏management / employer 🞏union 🞏association🞏police 🞏other, *please specify:__________*

**8.2.** If YES, what were the consequences for the perpetrators?

🞏 None 🞏 Verbal warning issued by hospital managers 🞏 Care discontinued

🞏 Reported to police 🞏 Aggressor prosecuted 🞏 Other:____ 🞏 Don't know

**C 9** Did your employer or supervisor offer to provide you with:

**9.1** Counseling 🞏 Yes 🞏 No

**9.2** Opportunity to speak about/report it 🞏 Yes 🞏 No

**9.3** Other support? 🞏 Yes 🞏 No

**C 10** How satisfied are you with the manner in which the incident was handled?*(Please rate: 1 = very dissatisfied, 5=very satisfied)*

🞏1 🞏2 🞏3 🞏4 🞏5

**C 11** If you did **not** report or tell about the incident to others, why not?

*(Please tick every relevant box)*

🞏 It was not important 🞏 Felt ashamed 🞏 Felt guilty

🞏 Afraid of negative consequences 🞏 Useless

🞏 Did not know who to report to 🞏 Other, *please specify*:__________

**D. HEALTH SECTOR EMPLOYER**

**D1** Has your employer developed specific policies on:

| Health and safety | 🞏 Yes | 🞏 No | 🞏 Don’t know |
| --- | --- | --- | --- |
| Physical workplace violence | 🞏 Yes | 🞏 No | 🞏 Don’t know |
| Verbal abuse | 🞏 Yes | 🞏 No | 🞏 Don’t know |
| Bullying/Mobbing | 🞏 Yes | 🞏 No | 🞏 Don’t know |
| Threat | 🞏 Yes | 🞏 No | 🞏 Don’t know |
| Sexual harassment | 🞏 Yes | 🞏 No | 🞏 Don’t know |

D2 Please choose the following measures for “The original” or “The new”. The new measures refer to those measures which were taken after serious violent incidents occurred. And assess these measures from “Very” to “Not at all”.

| Measures in detail | The  original | The  new | Policy measures effect assessment | | | |
| --- | --- | --- | --- | --- | --- | --- |
|  |  |  | Very | Moderate | Little | Not at  all |
| Security measures（e.g. guard, alarm device） | 🞏 | 🞏 | 🞏 | 🞏 | 🞏 | 🞏 |
| Police on duty room | 🞏 | 🞏 | 🞏 | 🞏 | 🞏 | 🞏 |
| Improve surroundings (e.g. lighting, noise, heat, access to food, cleanliness, privacy) | 🞏 | 🞏 | 🞏 | 🞏 | 🞏 | 🞏 |
| Restrict public access | 🞏 | 🞏 | 🞏 | 🞏 | 🞏 | 🞏 |
| Patient screening (to record and be aware of previous aggressive behaviour) | 🞏 | 🞏 | 🞏 | 🞏 | 🞏 | 🞏 |
| Patient protocols (e.g. control and restraint procedures, transport, medication, activities programming, access to information) | 🞏 | 🞏 | 🞏 | 🞏 | 🞏 | 🞏 |
| Increased staff numbers | 🞏 | 🞏 | 🞏 | 🞏 | 🞏 | 🞏 |
| Check-in procedures for staff | 🞏 | 🞏 | 🞏 | 🞏 | 🞏 | 🞏 |
| Special equipment or clothing (e.g. uniform or absence of uniform) | 🞏 | 🞏 | 🞏 | 🞏 | 🞏 | 🞏 |
| Changed shifts or rotas (i.e. working times) | 🞏 | 🞏 | 🞏 | 🞏 | 🞏 | 🞏 |
| Reduced periods of working alone | 🞏 | 🞏 | 🞏 | 🞏 | 🞏 | 🞏 |
| Training | 🞏 | 🞏 | 🞏 | 🞏 | 🞏 | 🞏 |
| None of these, Other ________ | 🞏 | 🞏 | 🞏 | 🞏 | 🞏 | 🞏 |
